# Spinal Excitability Changes after Transspinal and Transcortical Paired Associative Stimulation in Humans

**DOI:** 10.1155/2017/6751810

**Published:** 2017-10-16

**Authors:** Maria Knikou

**Affiliations:** ^1^Klab4Recovery, Department of Physical Therapy, College of Staten Island, New York, NY 10314, USA; ^2^Graduate Center, City University of New York, New York, NY 10016, USA

## Abstract

Paired associative stimulation (PAS) produces enduring neuroplasticity based on Hebbian associative plasticity. This study established the changes in spinal motoneuronal excitability by pairing transcortical and transspinal stimulation. Transcortical stimulation was delivered after (transspinal-transcortical PAS) or before (transcortical-transspinal PAS) transspinal stimulation. Before and after 40 minutes of each PAS protocol, spinal neural excitability was assessed based on the amplitude of the transspinal-evoked potentials (TEPs) recorded from ankle muscles of both legs at different stimulation intensities (recruitment input-output curve). Changes in TEPs amplitude in response to low-frequency stimulation and paired transspinal stimuli were also established before and after each PAS protocol. TEP recruitment input-output curves revealed a generalized depression of TEPs in most ankle muscles of both legs after both PAS protocols that coincided with an increased gain only after transcortical-transspinal PAS. Transcortical-transspinal PAS increased and transspinal-transcortical PAS decreased the low-frequency-dependent TEP depression, whereas neither PAS protocol affected the TEP depression observed upon paired transspinal stimuli. These findings support the notion that transspinal and transcortical PAS has the ability to alter concomitantly cortical and spinal synaptic activity. Transspinal and transcortical PAS may contribute to the development of rehabilitation strategies in people with bilateral increased motoneuronal excitability due to cortical or spinal lesions.

## 1. Introduction

Activity-dependent strengthening of synapses is an important mechanism underlying neural plasticity in both invertebrates and vertebrates [1]. In this context, human corticospinal excitability is affected by paired stimuli delivered to a mixed peripheral nerve of arms or legs and motor cortex based on the timing between the two associative stimuli [2–9]. For example, human corticospinal excitability increases when single electrical stimuli to a mixed peripheral nerve are followed by transcranial magnetic stimulation (TMS) at an interval that TMS and afferent volley-mediated inputs to the motor cortex occur simultaneously [7]. In contrast, corticospinal excitability decreases when the afferent volley from the periphery reaches the motor cortex after TMS [9]. These neuromodulatory effects have been ascribed to long-term potentiation (LTP) and long-term depression (LTD), resembling largely the spike-timing-dependent plasticity that has been described extensively in animal models [10].

Plasticity of cortical, corticospinal, and spinal human neural excitability as a result of paired associative stimulation (PAS) delivered to a mixed peripheral nerve and motor cortex is well established [8, 11–16]. However, neural excitability changes beyond the classical peripheral nerve-TMS PAS protocol have not been studied in detail. We have recently reported that transspinal and transcortical PAS produces distinct changes in neural excitability based on the timing between the two associative stimuli [17]. Specifically, intracortical inhibition decreases and intracortical facilitation and corticospinal excitability increase when TMS is delivered after transspinal stimulation (transspinal-transcortical PAS) [17]. In contrast, cortical feedback mechanisms remain unaltered, and corticospinal excitability decreases when TMS is delivered before transspinal stimulation (transcortical-transspinal PAS) [17]. These neuromodulatory effects coincided with directional changes of the excitability threshold of muscle spindle primary (Ia) afferents [17].

Based on these strong neuromodulatory effects, in this study we investigated to what extent transspinal and transcortical PAS alters the amplitude of transspinal-evoked potentials (TEPs) recorded from shank muscles of both legs. TEPs can be induced in nearly all muscles of both legs simultaneously upon electrical stimulation over the thoracolumbar region of the spine and have been associated with action potentials travelling in both antidromic and orthodromic directions along the posterior and anterior root fibers of both legs [18–20]. We hypothesized that transcortical-transspinal PAS increases spinal output by strengthening corticospinal synapses largely due to coincidence of the two stimuli on spinal alpha motoneurons. The rationale of this hypothesis was based on the fact that TMS and transspinal stimulation results in summation of tibialis anterior (TA) TEPs and motor-evoked potentials (MEPs) when these two stimuli interact at spinal level [17, 19]. We further hypothesized that transspinal-transcortical PAS either decreases or has no significant effects on spinal output. The rationale for this hypothesis is based on the fact that transspinal stimulation delivered before TMS has sufficient time to affect spinal neural circuits before descending motor volleys reach the spinal cord, while transspinal conditioning stimulation induces soleus H-reflex depression [18]. To test our hypotheses, we applied 40 minutes of associative stimulation to the thoracic spinal cord and motor cortex in healthy humans. We demonstrate a nondirectional PAS-mediated plasticity of TEPs and a directional PAS-mediated changes in TEPs frequency-dependent depression of several ankle muscles from both legs. These results constitute the first evidence for a novel PAS protocol that can be used to alter human motor pathways of both legs and may contribute to the development of rehabilitation strategies in people with bilateral increased spinal motoneuronal excitability.

## 2. Methods

### 2.1. Subjects

Fifteen subjects (5 females; all right leg dominant; mean age 23.67 ± 2.35 years; mean ± SD) without any history of neurological or musculoskeletal disorder or contraindication to TMS participated in the present study after providing written informed consent in accordance with the Declaration of Helsinki. The study was approved by the ethics committee of the City University of New York. A total of 27 experiments were completed on different days and were separated by a 3-week interval for the same subject. All subjects participated in a previous study [17], but data reported here are from a separate experimental session.

### 2.2. EMG Recordings

Surface electromyography (EMG) was recorded bilaterally from the TA, medial gastrocnemius (MG), soleus (SOL), and peroneus longus (PL) muscles via single bipolar differential electrodes (MA300-28, Motion Lab Systems Inc., Baton Rouge, LA). EMG signals were amplified, filtered (10–1000 Hz), sampled at 2000 Hz via a 1401 plus using Spike 2 software (Cambridge Electronics Design Ltd., England, UK), and stored for offline analysis.

### 2.3. Stimulation

#### 2.3.1. Transcortical Stimulation

TMS over the left primary motor cortex was delivered via a Magstim 200 stimulator (Magstim, UK) with a double-cone coil (diameter 110 mm), positioned such that the current flowed from a posterior to an anterior direction. The procedures were similar to those we have previously utilized [19, 21]. Briefly, with the double cone coil held at the motor hot spot, the stimulation intensity was gradually increased from zero, and MEPs recorded from the right TA and SOL muscles were observed on a digital oscilloscope (TBS1000, Tektronix). When at low stimulation intensities, MEPs in the right TA muscle could not be evoked without concomitant MEPs in the SOL muscle, the magnetic coil was moved, and the procedure was repeated. When the optimal position was determined, the right TA MEP resting threshold was established and corresponded to the lowest stimulation intensity that induced reproducible MEPs of at least ~50 *μ*V in 3 out of 5 consecutive single TMS pulses.

#### 2.3.2. Transspinal Stimulation

The Thoracic 10 spinous process was identified via palpation, and a single cathode electrode (Uni-Patch EP84169, 10.2 cm ± 5.1 cm, Wabasha, MA) was placed along the vertebrae equally between the left and right paravertebral sides. Due to its size, the electrode covered from Thoracic 10 to Lumbar 1-2 vertebral levels. These vertebral levels correspond to spinal segments and segmental innervation of the muscles from which compound muscle action potentials were recorded in this study. Two reusable self-adhered electrodes (anode; same type as the cathode), connected to function as a single electrode, were placed bilaterally on the abdominal muscles or iliac crests based on self-reported discomfort by the subjects upon transspinal stimulation. The cathode and anode electrodes were connected to a constant current stimulator (DS7A, Digitimer, UK) that was triggered by Spike 2 scripts (CED Ltd., UK).

### 2.4. Transspinal and Transcortical PAS

Paired stimulation included 240 pairs of TMS pulses delivered over the area of the motor cortex corresponding to the right TA muscle and cathodal transspinal stimulation delivered to the spine. Both paired stimuli were delivered at 0.1 Hz for 40 minutes with subjects supine. Knee and hip joints were flexed at 30° and ankles were in neutral position.

**Figure 1:**
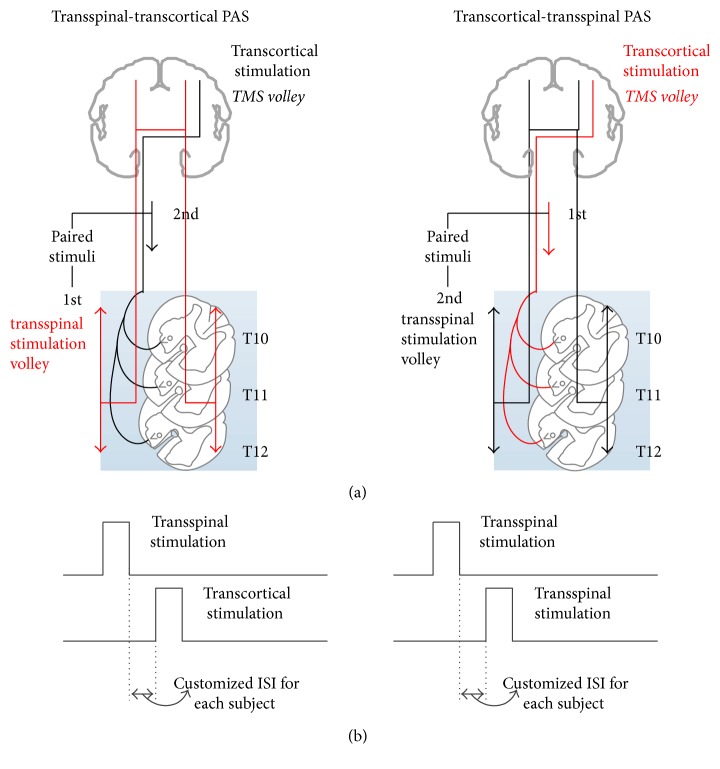
Paired associative stimulation (PAS) protocol. (a) Simplified diagram of transcranial magnetic stimulation (TMS volley) and transspinal stimulation-mediated volleys during paired stimulation. TMS motor volleys are descending, whilst transspinal stimulation produces both ascending and descending volleys. The ascending volleys are expected to reach both brain hemispheres since transspinal stimulation delivered alone evokes transspinal-evoked potentials (TEPs) in muscles of both legs. (b) Timing of PAS between transcortical and transspinal stimulation. PAS was delivered at customized interstimulus intervals for each subject during which corticospinal neurons activated via TMS arrived at the corticospinal neuron before spinal motoneurons were activated transsynaptically by the transspinal stimulation (transcortical-transspinal PAS), and during which transspinal-mediated ascending volleys arrived at the motor cortex before TMS was delivered over the left primary motor cortex leg area (transspinal-transcortical PAS).

The interstimulus interval (ISI) between transspinal and transcortical stimuli during PAS was customized for each subject and was identical to that we have previously utilized to establish cortical, corticospinal, and soleus Ia afferents excitability changes after transspinal and transcortical PAS [17]. The ISI for each subject was estimated using the onset latencies of the TA EMG responses to TMS (TA MEP) and transspinal stimulation (TA TEP). The conduction time from the motor cortex to corticospinal presynaptic terminals was estimated by adding 1.5 ms to the TA TEP latency, and the resultant value was subtracted from the TA MEP latency (1). The added 1.5 ms is the time required for synaptic transmission and conduction to the lumbar nerve root at the vertebral foramina [2, 8]. This calculation resulted in ISIs that ranged from 8 to 13.5 ms (10.82 ± 0.56 ms) across subjects. The same customized ISI was used for each subject during PAS, while reversing the onset of stimuli with respect to transspinal and transcortical stimulation (Figure 1).

(1) $$\begin{array}{c} ISI=TA\;MEP\;latency-\left(TA\;TEP\;latency+1.5\right). \end{array}$$

The ISIs used allowed transspinal stimulation to evoke depolarization of spinal motoneurons before descending motor volleys arrived at the presynaptic terminals of corticospinal neurons in the transspinal-transcortical PAS protocol (Figure 1(a)). Further, transspinal stimulation at intensities sufficient to evoke TEPs in both leg muscles produces ascending and descending volleys from muscle and cutaneous afferents. At the ISIs used, transspinal-induced ascending volleys reached the primary motor cortex at times sufficient to affect descending motor volleys at the cortical level. This is supported by the timing of distal limb afferent-mediated TA MEP facilitation [22–24] and onset latency (10 to 15 ms) of cortical potentials induced via thoracic transspinal stimulation [25]. Thus, in the transspinal-transcortical PAS protocol, both stimuli likely interacted at cortical level. Additionally, the ISIs used allowed descending motor volleys elicited by TMS to arrive at the presynaptic terminals of corticospinal neurons before transspinal stimulation transsynaptically evoked depolarization in spinal alpha motoneurons in the transcortical-transspinal PAS protocol (Figure 1(a)). Consequently, in the transcortical-transspinal PAS protocol, both stimuli likely interacted at spinal level. The latter is further supported by the central spinal conduction time (10.5 ± 0.9 ms) of the TA MEP [26].

In the transspinal-transcortical PAS protocol, TMS was delivered at 1.13 ± 0.02 (63.42 ± 1.26 maximum stimulator output (MSO)) of the right TA MEP resting threshold, and transspinal stimulation was delivered at 1.08 ± 0.05 (48.38 ± 3.9 mA) of the right TA TEP threshold. In the transcortical-transspinal PAS protocol, TMS was delivered at 1.08 ± 0.02 (60.24 ± 2.29 MSO) of the right TA MEP resting threshold and transspinal stimulation was delivered at 1.14 ± 0.05 (54.6 ± 3.58 mA) of the right TA TEP threshold. Both transspinal and transcortical stimuli during PAS were set to produce similar in amplitudes right TA TEPs and MEPs, which were evoked on the ascending portion of the associated recruitment input/output curve and were equivalent to 30% of the associated maximal values. However, adjustments were made based on self-reported discomfort by the subjects.

### 2.5. Neurophysiological Recordings before and after PAS

With subjects supine, changes in spinal excitability were assessed based on the recruitment input-output TEP curve of ankle muscles before and after transcortical-transspinal (*N* = 15) and transspinal-transcortical (*N* = 12) PAS. Transspinal stimulation was delivered at 0.2 Hz, and at least 120 responses were recorded at different stimulation intensities, starting from below TEP threshold for all ankle muscles until maximal amplitudes were evoked. Four responses were recorded at each stimulation intensity. TEPs were recorded from synergistic/antagonistic ankle muscles of both legs, because TMS at ~1.2 MEP resting threshold creates multiple descending volleys, activating corticomotoneuronal cells projecting to agonist and antagonist motoneurons and spinal inhibitory interneurons [27, 28].

In order to establish whether spinal excitability changes were mediated at the presynaptic level, 15 TEPs elicited at 0.2 Hz and at 1.0 Hz stimulation frequencies were recorded. This approach was selected based on the well-documented decrease in efficiency of individual synapses when repeatedly activated at low frequencies, ascribed to transmitter substance depletion at the presynaptic terminals [29]. To further assess changes in spinal excitability, TEPs were also recorded in response to paired stimuli at 50 and 100 ms ISIs before and after each PAS protocol. TEPs recorded at different frequencies and upon paired pulses were evoked at the ascending portion of the TEP recruitment curve (1.2 TA TEP threshold) across subjects, but at the same stimulation intensity before and after each PAS protocol for each subject.

### 2.6. Data Analysis and Statistics

TEPs from all muscles were measured as the area of the full-wave rectified EMG signal, because the area under the curve detects accurately the inhibition of compound muscle action potentials [30]. TEPs recorded at different stimulation intensities (recruitment curve) were normalized to the homonymous maximal TEP observed before PAS. The stimulation intensities were normalized to the intensity required to elicit a TEP equivalent to 50% of TEPmax (S50) before PAS. The S50 was estimated based on the sigmoid function fitted to the recruitment curve (see below). Then, the average normalized TEP was calculated in incremented steps of 0.05 of S50 for each subject and across subjects. Data was subjected to Shapiro-Wilk test for normal distribution, and a 2-way repeated measures analysis of variance (rmANOVA) was performed to determine the main effects of time on TEPs amplitude recorded at different stimulation intensities (recruitment curves). This analysis was done separately for each muscle from which TEPs were recorded and PAS protocol. When a statistically significant difference was found, Bonferroni or Holm-Sidak post hoc multiples comparisons were performed.

A Boltzmann sigmoid function (2) (SigmaPlot 11, Systat Software Inc.) was fitted separately to the right and left SOL, TA, MG, and PL TEPs normalized to the homonymous maximal TEP observed before PAS and plotted against the nonnormalized (actual) stimulation intensities for each subject [19, 31]. The parameters in (2) represent the upper limb of the TEP recruitment curve to the point that the TEP amplitude is maximal (TEPmax), the slope parameter of the function *m*, the S50, and the TEP amplitude at a given stimulus value (TEP(*s*)). The slope of curve was constrained to occur at a stimulus intensity equivalent to S50 and was defined by the relationship indicated in (3). The stimuli corresponding to threshold and maximal TEP amplitudes were estimated based on (4) and (5), respectively. These sigmoid function predicted parameters were grouped across subjects based on time of testing, PAS protocol, and muscle. Data was subjected to Shapiro-Wilk test for normal distribution, and a 3-way rmANOVA was applied to the data to determine the main effects of time, PAS protocol, and muscle on the predicted maximal TEP amplitude and slope. When statistically significant differences were found, post hoc Bonferroni *t*-tests were performed.

(2) $$\begin{array}{c} TEP\left(s\right)=\frac{TEPmax}{\left(1+\text{exp}\left(m\left(S50-s\right)\right)\right)\;}, \end{array}$$

(3) $$\begin{array}{c} TEPslope=\frac{m\times TEPmax}{4}, \end{array}$$

(4) $$\begin{array}{c} TEPth\;stim=\frac{s-2}{m}, \end{array}$$

(5) $$\begin{array}{c} TEPmax\;stim=\frac{s+2}{m}. \end{array}$$

For the low-frequency TEP depression, the percent of change in TEP amplitudes elicited at 0.2 Hz from those elicited at 1.0 Hz was calculated for recordings taken before and after each PAS protocol. A two-way rmANOVA was performed to determine the main effects of PAS and time of testing on the percent of change of TEPs recorded at 1.0 Hz.

For the TEP depression induced upon paired transspinal stimuli, the mean size of the TEP evoked by the second pulse (TEP_2_) was normalized to the mean size of the homonymous TEP evoked by the first pulse (TEP_1_). This was done separately for each subject, muscle from which TEPs were recorded, and time of testing. Then, the normalized TEPs were grouped based on muscle, time of testing, and PAS protocol for each ISI (50 and 100 ms) tested. A 3-way rmANOVA was applied to the data to establish statistically significant differences among these factors for each ISI tested. Data are presented as mean ± SE.

## 3. Results

### 3.1. Changes in Spinal Excitability after Transspinal and Transcortical PAS

**Figure 2:**
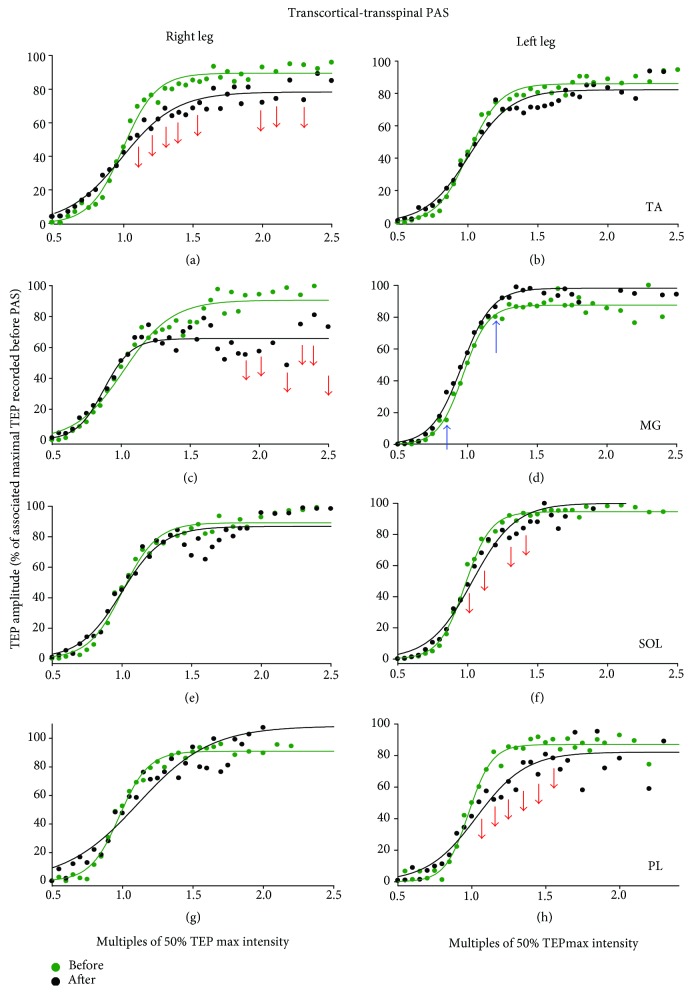
TEPs recruitment curves before and after transcortical-transspinal PAS. Recruitment input-output curves of transspinal-evoked potentials (TEPs) recorded bilaterally from the TA, MG, SOL, and PL muscles from all subjects along with the sigmoid function fitted to the data. The abscissa shows multiples of stimulation intensities corresponding to 50% TEP max (S50). The ordinate shows TEP sizes as a percentage of the homonymous maximal TEP size obtained before transcortical-transspinal PAS. Red or blue arrows indicate statistically significant differences (decreased and increased amplitudes, resp.) before and after PAS based on 2-way repeated measures ANOVA. TA: tibialis anterior; MG: medial gastrocnemius; SOL: soleus; PL: peroneus longus.

The TEP recruitment input-output curves from all subjects and muscles recorded before and after *transcortical-transspinal PAS* are indicated in Figure 2. A 2-way rmANOVA with main factors time and stimulation intensities normalized to S50 showed a significant effect of time (*F*_(1)_ = 11.81, *p* < 0.001) for the right TA TEP (Figure 2(a)), while significant interactions between time and intensities were found (*F*_(24)_ = 1.62, *p* = 0.034). Significant main time effects were also found for the right MG, left MG, left SOL, and left PL TEPs (*p* < 0.001 for all). The results of Holm-Sidak pairwise multiple comparisons before and after transcortical-transspinal PAS are indicated as arrows in Figure 2. These findings suggest that transcortical-transspinal PAS decreased spinal excitability, but this decrease was not distributed uniformly in the TEPs of both legs.

**Figure 3:**
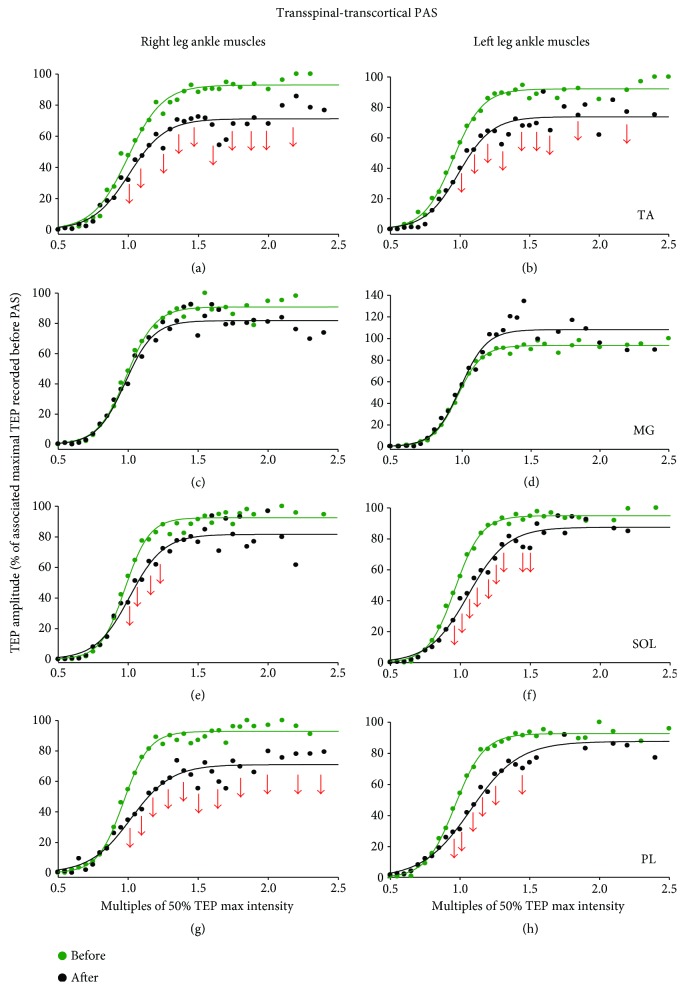
TEPs recruitment curves before and after transspinal-transcortical PAS. Recruitment input-output curves of transspinal-evoked potentials (TEPs) recorded bilaterally from the TA, MG, SOL, and PL muscles from all subjects along with the sigmoid function fitted to the data. The abscissa shows multiples of stimulation intensities corresponding to 50% TEP max (S50). The ordinate shows TEP sizes as a percentage of the homonymous maximal TEP size obtained before transspinal-transcortical PAS. Red arrows indicate statistically significant differences (decreased amplitudes) before and after PAS based on repeated measures ANOVA. TA: tibialis anterior; MG: medial gastrocnemius; SOL: soleus; PL: peroneus longus.

The TEP recruitment input-output curves from all subjects and muscles recorded before and after *transspinal-transcortical PAS* are indicated in Figure 3. A 2-way rmANOVA with main factors time and stimulation intensities normalized to S50 showed a significant effect of time (*F*_(1)_ = 69.38, *p* < 0.001) for the right TA TEP (Figure 3(a)), while significant interactions between time and intensities were found (*F*_(24)_ = 2.04, *p* = 0.003). Significant main time effects were also found for the left TA, left MG, right/left SOL, and right/left PL TEPs (*p* < 0.001 for all). A significant interaction between time and stimulation intensities normalized to S50 was found for the right/left TA, right/left SOL, and right PL TEPs (all *p* < 0.05). The results of Holm-Sidak pairwise multiple comparisons before and after transspinal-transcortical PAS are indicated as arrows in Figure 3. These findings suggest that transspinal-transcortical PAS decreased most (6 out of 8) of the ankle TEPs.

The estimated parameters from the sigmoid function fitted to the TEP recruitment input-output curves are shown in Tables 1 and 2. For the predicted maximal TEP amplitudes, rmANOVA with main factors time, PAS protocol, and muscle showed a significant effect of time (*F*_(1)_ = 7.73, *p* = 0.006) and muscle (*F*_(7)_ = 2.57, *p* = 0.013) and a nonsignificant effect between PAS protocols (*F*_(1)_ = 0.45, *p* = 0.5). Statistically significant interactions between muscle and time (*F*_(7)_ = 2.59, *p* = 0.013) and between PAS protocols and time (*F*_(1)_ = 6.55, *p* = 0.011) for the predicted maximal TEP amplitudes were found. Further, for the predicted slope of the TEPs input/output curves, rmANOVA with main factors time, PAS protocol, and muscle showed a statistically significant effect of time (*F*_(1)_ = 19.67, *p* < 0.001) and PAS protocol (*F*_(1)_ = 6.99, *p* = 0.009) but not between muscles from which TEPs were recorded (*F*_(7)_ = 0.9, *p* = 0.5). Statistically significant interactions were found only between PAS protocols and time (*F*_(1)_ = 8.2, *p* = 0.004) for the predicted slope of the TEP curve. These findings suggest that transcortical-transspinal PAS increases the gain of input-output relationship differently across various motoneurons.

### 3.2. Changes in Spinal Inhibition after Transspinal and Transcortical PAS

**Figure 4:**
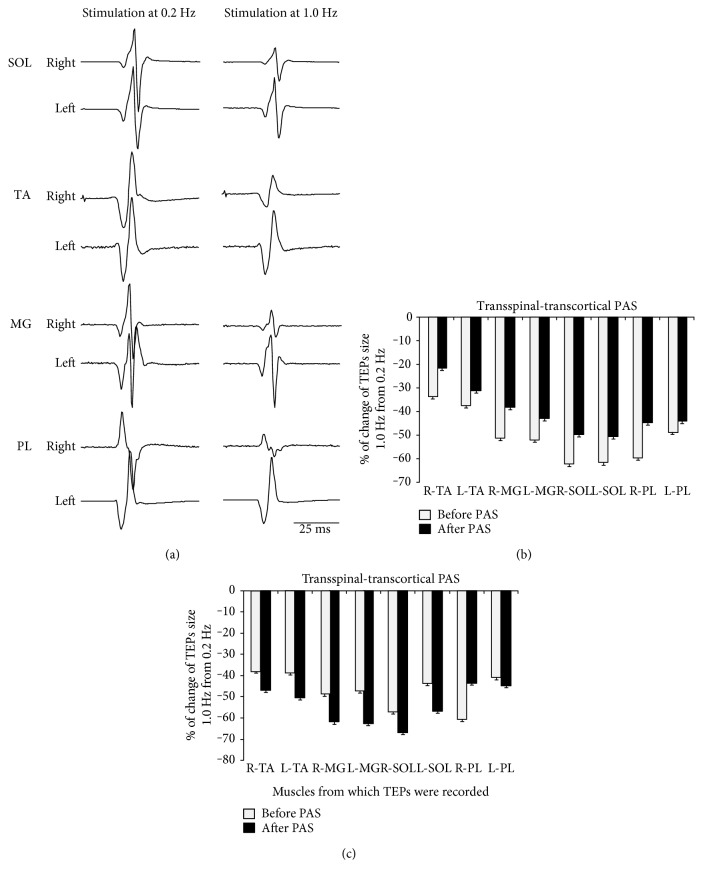
Frequency-dependent depression of TEPs. (a) Nonrectified waveform averages of ankle transspinal-evoked potentials (TEPs) recorded from one representative subject at 0.2 and 1.0 Hz. (b, c) Overall percent change of TEPs recorded at 0.2 Hz from the associated TEP recorded at 1.0 Hz before and after each PAS protocol from all subjects. The abscissa shows the muscles from which TEPs were recorded. Error bars indicate SE.

Figure 4(a) shows waveform averages of SOL, TA, MG, and PL TEPs from one participant evoked at 0.2 Hz and 1.0 Hz. In this participant, TEP frequency-dependent depression was not distributed equally between the right and left legs (Figure 4(a)). The TEP frequency-dependent depression is easily recognized by the overall percent change observed when TEPs were evoked at 1.0 Hz from 0.2 Hz before transcortical-transspinal (Figure 4(b)) and transspinal-transcortical (Figure 4(c)) PAS. It is evident that TEPs evoked at 1.0 Hz were reduced by as much as 70% from their respective TEPs evoked at 0.2 Hz. rmANOVA with the main factors PAS protocols, muscles, and time showed that the percent of change of TEPs recorded at 1.0 Hz was significantly different across muscles (*F*_(7)_ = 3.53, *p* < 0.001), but not between PAS protocols (*F*_(1)_ = 2.5, *p* = 0.11) or time of testing (*F*_(1)_ = 0.22, *p* = 0.63). Statistically significant interactions between PAS protocols and time of testing were found (*F*_(15)_ = 8.09, *p* = 0.005). Specifically, pairwise multiple comparisons showed that the right TA (*t* = 2.48, *p* = 0.013) and left SOL (*t* = 2.21, *p* = 0.027) low-frequency TEP depression was different between the two PAS protocols. Further, the right MG (*t* = 2.23, *p* = 0.026) and left PL (*t* = 2.32, *p* = 0.021) TEP sizes were significantly different as a function of time. A significant effect was found for TEPs recorded before and after transspinal-transcortical PAS (*t* = 3.12, *p* = 0.002).

**Figure 5:**
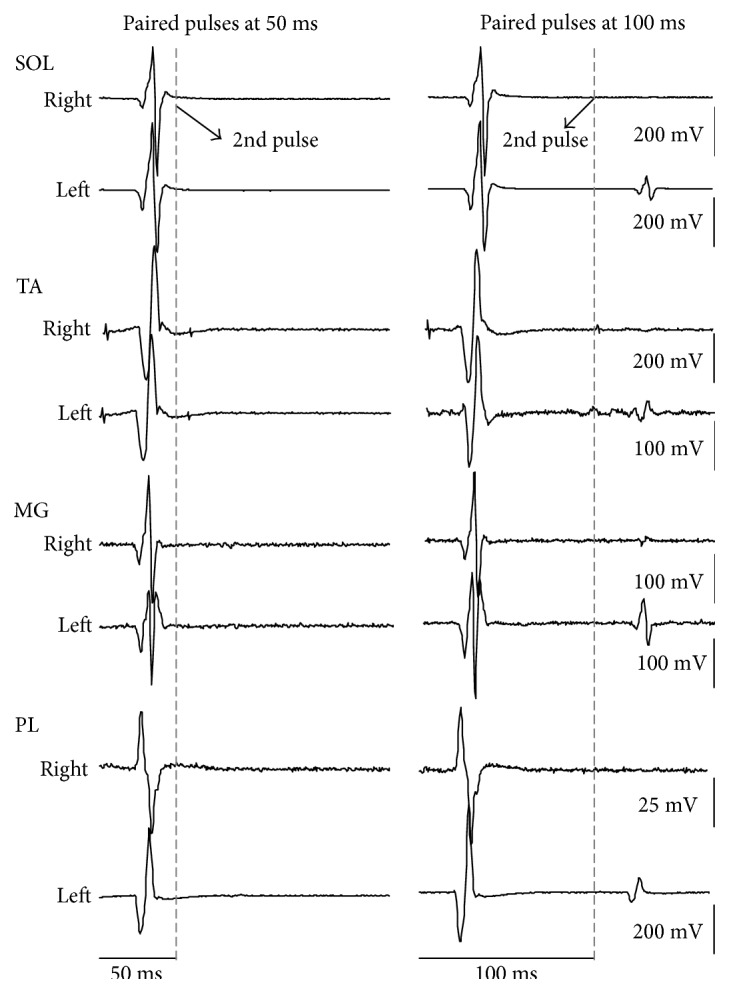
Depression of TEPs in response to paired transspinal stimuli. Nonrectified waveform averages of ankle transspinal-evoked potentials (TEPs) recorded from one representative subject upon paired pulses at interstimulus intervals of 50 and 100 ms at a constant stimulation frequency of 0.2 Hz.

Waveform averages of SOL, TA, MG, and PL TEPs from one participant recorded in response to paired transspinal stimuli at ISIs of 50 and 100 ms from both legs are depicted in Figure 5. It is apparent that the TEPs recorded from extensor and flexor ankle muscles of both legs were significantly reduced upon paired transspinal stimuli pulses.

**Figure 6:**
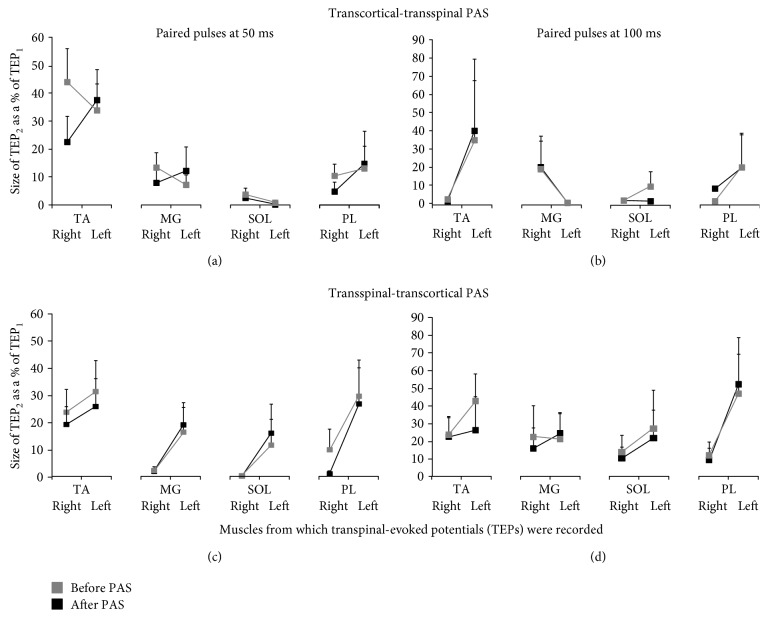
Depression of TEPs in response to paired transspinal stimuli before and after transspinal and transcortical PAS. Overall amplitude of TEP_2_ as a percentage of the mean TEP_1_ evoked at interstimulus intervals of 50 (a, c) and 100 ms (b, d) at a stimulation frequency of 0.2 Hz before and after each PAS protocol from all subjects. The abscissa shows the muscles from which TEPs were recorded. Error bars indicate SE. No changes in TEPs depression upon paired pulses before and after transspinal and transcortical PAS were found.

Figure 6 shows the overall mean amplitude of TEP_2_ (% of mean TEP_1_) elicited by a pulse delivered 50 or 100 ms after TEP_1_. For TEPs recorded upon paired stimuli at 50 ms, rmANOVA with the main factors PAS protocols, time, and muscles from which TEPs were recorded showed a significant effect between muscles (*F*_(7)_ = 8.21, *p* < 0.001) but not between PAS protocols (*F*_(1)_ = 0.04, *p* = 0.84) or time (*F*_(1)_ = 0.74, *p* = 0.39). Similar results were also observed for TEPs recorded following paired transspinal stimuli at an ISI of 100 ms. These findings suggest that the TEP depression evoked upon paired stimuli was not the same among muscles and was not affected by either PAS protocol.

## 4. Discussion

This study demonstrates that PAS-induced plasticity of different spinal motoneurons for both legs can be achieved by pairing transspinal and transcortical stimulation. This new PAS protocol produced an overall depression of spinal motor output and may constitute a promising neuromodulatory paradigm especially for people with increased bilateral neural excitability.

The excitability of most ankle flexor/extensor muscles from both legs, based on the recruitment TEP input-output curves, was reduced regardless of the PAS protocol (Figures 2 and 3). The decreased TEPs amplitude after both PAS protocols is in contrast to the well-documented timing-dependent plasticity and synapse-specific following peripheral nerve-TMS PAS in humans [32]. For example, 15 min of paired TMS followed by posterior tibial nerve stimulation at an ISI of 20 ms results in soleus H-reflex facilitation and decreased threshold of soleus Ia afferents [33]. The different effects may be related to the neural elements being excited following stimulation. Low-intensity stimulation of a mixed peripheral nerve selectively excites muscle spindle Ia afferents, while transspinal stimulation excites fibers of the spinal cord at their entry to or exit from the spinal canal generating action potentials that travel antidromic and orthodromic along the posterior and anterior root fibers bilateral [34]. Further, excitation of motor axons and muscle spindle afferents and all of their terminal branches lead to transsynaptic excitation of motoneurons and interneurons near and far from the stimulation site [18, 34–36]. These results support the notion that transspinal stimulation is more complex in nature compared to a single stimulus delivered to a mixed peripheral nerve, and thus differences of findings between peripheral-TMS PAS and transspinal-transcortical PAS can be readily attributed to dissimilar functional network interactions between the two associative inputs.

The site of TEPs depression after transspinal-transcortical PAS is likely cortical, because transspinal stimulation, through the dorsal spinocerebellar and large diameter dorsal column fibers [37], can modulate activity of cortical feedback mechanisms altering the strength of TMS-induced descending motor volleys at their origin site decreasing thereafter spinal output. The unaltered slope of the TEPs input-output relationship after transspinal-transcortical PAS supports further that the transspinal-induced ascending volleys interacted at the cortical level leaving unchanged the gain of spinal motoneurons after PAS. Cortico-cortical interneuronal circuits involving low-threshold *γ*-aminobutyric acid receptor-dependent inhibitory pathways [38] may account partly for the TEPs depression after transspinal-transcortical PAS, since reduced intracortical inhibition constitutes one of the mechanisms of MEP modulation after peripheral nerve-TMS PAS [39]. Specifically, GABA_A_ receptor-mediated short-interval intracortical inhibition blocks the MEP facilitation produced by peripheral nerve-TMS PAS [40], while PAS-mediated effects depend largely on interactions between cortical circuits on the basis that short-latency afferent inhibition determines the efficacy of PAS [41]. Further, interhemispheric inhibition, which is affected by PAS [42], can result in LTD-like effects during peripheral nerve-TMS PAS [43]. In addition, because transspinal stimulation reached both hemispheres, the decreased TEPs amplitude after transspinal-transcortical PAS may be related to the phenomenon of surround organization whereas response properties of neurons are changed by stimulation outside the neuron's classical receptive field [44, 45]. Last, when comparing the decreased spinal excitability found in this study with that of increased corticospinal excitability after transspinal-transcortical PAS and peripheral nerve-TMS PAS [7, 16, 17], we can conclude that cortical and spinal plasticity after transspinal-transcortical PAS occurs in diametrical opposite directions, a phenomenon that has great potential in neurological disorders with decreased corticospinal drive and increased spinal motoneuronal excitability as is the case in cerebral and spinal lesions. These findings support the notion that transspinal-transcortical PAS effects involved motor cortices, but investigation of changes in cortical circuits warrants further investigation.

The site of TEPs depression after transcortical-transspinal PAS is likely spinal, based on the relative conduction velocities and interactions of MEPs and TEPs [17, 19], and decreased soleus H-reflex excitability upon transspinal conditioning stimulation [18]. LTD of synaptic efficacy may account for depression of TEPs after transcortical-transspinal PAS, because LTD requires postsynaptic spikes leading presynaptic spikes [46–49]. This is consistent with the transcortical-transspinal PAS, during which transcortical stimulation produced presynaptic volleys to spinal motoneurons, and transspinal stimulation produced antidromic postsynaptic volleys. LTD likely occurred at the synapses connecting corticospinal neurons with alpha motoneurons decreasing the overall transsynaptic activation of motoneurons induced by transspinal stimulation. Because long-lasting voltage-dependent calcium channels are necessary for LTD, we can suggest that plateau potentials through changes of homeostatic mechanisms controlling background network activity level contributed to TEPs depression [32, 50]. The increased TEP slope (Table 1), which is believed to reflect changes in recruitment gain or transsynaptic excitability [51, 52] along with the altered stimulation intensities at maximal intensities for some TEPs, supports further the involvement of plateau potentials. Plateau potentials can change the relationship between input and firing rate [53], compress the range of thresholds, and alter the input-output relationship [54]. Another possible mechanism is that of changes in activity of cortical interneurons on corticomotoneuronal cells affecting the size of subliminal fringe of both cortical and spinal neural cells. This mechanism is supported by the decreased TA MEP input/output curve and increased curve slope after transcortical-transspinal PAS [17]. Nonetheless, *in vivo* and *in vitro* studies are needed to explore the physiological and pharmacological mechanisms involved in depression of spinal output after transcortical-transspinal PAS.

In this study, transcortical-transspinal PAS increased and transspinal-transcortical PAS decreased the frequency-dependent TEP depression in the right TA/MG and left SOL/PL TEPs (compare Figures 4(b) and 4(c)). These findings support directional changes in the mechanism directly coupled to the release process of neurotransmitters at the presynaptic terminals [29]. Furthermore, these findings support the notion that the effects were distributed simultaneously in multiple afferent-motoneuron synapses. Our current results are consistent with the reduction of soleus H-reflex frequency-dependent depression after transspinal-transcortical PAS [17], and reduced presynaptic inhibition of Ia afferent fibers after peripheral nerve-TMS PAS [13]. Although it is not possible to make certain conclusions regarding the exact mechanism of the observed opposite effects on Ia afferent-motoneuron synapses following PAS, certain possibilities may be considered. PAS-induced plastic changes in the motor cortex could have potentially affected the tonic discharge of cortical neurons resulting in altered corticospinal control exerted on Ia afferent-motoneuron synapses bilaterally [55]. This mechanism can readily account for the changes observed after transspinal-transcortical PAS. In contrast, the changes observed following transcortical-transspinal PAS can potentially result from potentiation of activity of ipsilateral spinal inhibitory interneurons and commissural interneurons, but further research is needed.

We further theorized that the frequency-dependent and paired transspinal stimuli-induced TEP depression would be modulated in a similar manner after PAS. However, the paired transspinal stimuli-induced TEP depression was not affected by transcortical-transspinal or transspinal-transcortical PAS (Figure 6). At this point, we should consider whether the TEP depression in response to low frequency and paired stimuli is mediated by similar neural circuits or mechanisms [29, 56, 57]. The TEPs depression upon paired transspinal stimuli may be partly due to postsynaptic Ib inhibition or reciprocal Ia inhibition. This is supported by the bilateral contraction of leg muscles at intensities sufficient to induce TEPs. Thus, contraction-induced group I afferent discharges of multiple afferent terminals that synapse with spinal interneurons [29], such as Ia and Ib inhibitory interneurons, may contribute to the TEP depression. Studies on the neurophysiological properties of TEPs relative to different spinal inhibitory circuits are lacking; however, it is apparent that transcortical-transspinal and transspinal-transcortical PAS have distinct effects on different types of spinal inhibitory mechanisms.

PAS protocols assessing cortical and/or spinal neuromodulation after peripheral-TMS PAS have traditionally recorded changes from the targeted muscle [10]. In this study, we recorded changes bilaterally from synergistic and antagonistic muscles in order to delineate the effects of our novel PAS protocol on cortical and spinal synapses controlling output of multiple leg muscles. The TEP depression after transspinal-transcortical and transcortical-transspinal PAS was apparent not only in the right TA muscle, targeted by the TMS, but also in synergistic and antagonistic muscles of the ipsilateral and contralateral legs (Figures 2 and 3). The TEP depression after transspinal-transcortical PAS was distributed to the same muscles in the right and left legs (Figure 3). In contrast, the TEP depression after transcortical-transspinal PAS was not distributed equally between muscles, since the decreased right TA/MG TEPs amplitude did not coincide with decreased left TA/MG TEPs (Figure 2). Similarly, although a similar effect on low-frequency TEP depression was evident after both PAS protocols, the effect was not of equivalent strength between muscles (Figure 4 and Results). Taken altogether, this study for the first time has provided evidence that PAS can produce simultaneously different changes in many synapses at cortical and spinal levels.

## 5. Limitations of the Study

In this study, PAS was delivered in a single session for 40 minutes. Because the neurophysiological tests were not conducted at different times after PAS termination, and multiples sessions of PAS were not delivered, future studies are needed to assess the time course of the effects incorporating multiple sessions of this new PAS protocol. Furthermore, for both PAS protocols, single 1-ms pulses at 0.1 Hz were used based on previous human and animal studies [16, 58]. It is known that PAS-induced plasticity depends on the frequency of pulse trains [47, 59]. While the application of PAS at a low-frequency might have reduced the strength of plasticity, it cannot account for the neurophysiological differences we observed between the two PAS protocols.

## 6. Conclusion

Pairing transspinal and transcortical stimulation alters the excitability of different motoneuron pools of both legs in healthy humans. TEP recruitment input/output curves revealed a generalized depression in most ankle TEPs after both PAS protocols, while a directional-dependent PAS effect was found for the frequency-dependent TEP depression. These findings support the notion that transspinal and transcortical PAS has the ability to alter activity of multiple cortical and spinal synapses, and thus it may contribute to the development of rehabilitation strategies in people with bilateral increased motoneuronal excitability due to cortical or spinal lesions.

## Figures and Tables

**Table 1:** TEPs sigmoid function parameters before and after transcortical-transspinal PAS.

	R-TA		L-TA		R-MG		L-MG		R-SOL		L-SOL		R-PL		L-PL	
	Before	After	Before	After	Before	After	Before	After	Before	After	Before	After	Before	After	Before	After
Max amplitude	*93.97 ± 2.97*	*85.94 ± 8.36*	88.20 ± 1.55	94.87 ± 6.38	96.54 ± 5.60	91.20 ± 7.93	96.71 ± 1.55	103.95 ± 5.82	94.29 ± 1.56	95.26 ± 7.64	94.60 ± 0.78	103.9 ± 13.63	90.94 ± 0.93	92.06 ± 7.21	*95.41 ± 1.73*	*73.07 ± 8.40*
*m*	0.24 ± 0.06	0.32 ± 0.13	*0.31 ± 0.08*	*0.15 ± 0.03*	0.15 ± 0.02	0.13 ± 0.03	*0.22 ± 0.03*	*0.16 ± 0.02*	0.22 ± 0.03	0.17 ± 0.03	*0.23 ± 0.03*	*0.16 ± 0.02*	0.19 ± 0.02	0.14 ± 0.03	*0.27 ± 0.05*	*0.14 ± 0.03*
Slope	14.59 ± 2.56	20.57 ± 4.95	*10.28 ± 1.47*	*18.77 ± 2.87*	*17.85 ± 2.99*	*19.64 ± 2.18*	*11.37 ± 1.37*	*14.86 ± 1.61*	*12.45 ± 2.09*	*18.43 ± 3.02*	*10.56 ± 1.22*	*16.75 ± 2.83*	*10.98 ± 1.18*	*19.98 ± 4.78*	*9.78 ± 2.40*	*17.01 ± 2.44*
Stimulus threshold	40.50 ± 3.92	34.80 ± 4.38	44.78 ± 3.33	41.70 ± 3.68	42.79 ± 3.99	40.46 ± 5.66	44.87 ± 3.06	44.28 ± 3.19	43.32 ± 3.96	43.08 ± 5.23	46.49 ± 3.23	45.10 ± 3.11	40.12 ± 4.73	34.79 ± 7.96	39.70 ± 4.27	35.50 ± 5.60
Stimulus, 50% max	55.09 ± 4.19	55.29 ± 5.32	55.06 ± 3.79	60.46 ± 4.43	60.64 ± 4.67	60.10 ± 4.77	56.24 ± 3.78	59.14 ± 3.80	55.77 ± 4.53	61.51 ± 4.81	57.06 ± 4.05	61.85 ± 4.29	51.09 ± 4.99	54.77 ± 4.33	49.47 ± 4.15	52.52 ± 5.57
Stimulus max	69.97 ± 5.72	75.76 ± 9.29	*65.34 ± 4.69*	*79.23 ± 6.49*	78.48 ± 6.75	79.74 ± 4.81	67.61 ± 4.79	74.01 ± 4.89	68.22 ± 5.84	79.94 ± 6.09	67.62 ± 5.03	78.61 ± 6.58	*62.07 ± 5.50*	*74.75 ± 4.44*	59.25 ± 5.26	69.53 ± 6.52

Results of predicted parameters from the sigmoid input-output relation of TEPs recorded at all points of the recruitment curve. TEPs were normalized to the homonymous maximal TEP recorded before PAS and were plotted against real stimulation intensities. This was performed separately for each subject, and averages were estimated and grouped based on time. *m*: slope parameter of the function. Mean ± SE. TA: tibialis anterior; MG: medial gastrocnemius; SOL: soleus; PL: peroneus longus; R: right; L: left. Italics indicate statistically significant differences before and after PAS.

**Table 2:** TEPs sigmoid function parameters before and after transspinal-transcortical PAS.

	R-TA		L-TA		R-MG		L-MG		R-SOL		L-SOL		R-PL		L-PL	
	Before	After	Before	After	Before	After	Before	After	Before	After	Before	After	Before	After	Before	After
Max amplitude	*97.76 ± 0.75*	*73.87 ± 4.66*	*101.21 ± 2.89*	*75.19 ± 5.52*	96.60 ± 1.63	90.86 ± 7.71	99.63 ± 2.39	121.90 ± 22.9	*94.79 ± 1.24*	*82.60 ± 4.12*	*98.60 ± 1.70*	*77.16 ± 6.76*	*96.67 ± 2.48*	*70.04 ± 5.79*	*97.32 ± 1.62*	*83.83 ± 7.40*
*m*	0.18 ± 0.02	0.17 ± 0.02	0.20 ± 0.07	0.29 ± 0.15	0.23 ± 0.04	0.21 ± 0.04	0.24 ± 0.05	0.17 ± 0.01	0.23 ± 0.03	0.20 ± 0.03	0.20 ± 0.03	0.19 ± 0.03	0.20 ± 0.02	0.17 ± 0.02	0.17 ± 0.02	0.15 ± 0.02
Slope	12.97 ± 1.46	13.44 ± 1.43	15.79 ± 2.22	14.38 ± 2.31	11.33 ± 1.65	12.42 ± 1.69	11.12 ± 1.42	12.96 ± 3.42	10.06 ± 1.31	11.70 ± 1.38	12.17 ± 1.51	13.24 ± 1.81	12.24 ± 1.87	13.89 ± 1.53	13.62 ± 1.45	17.63 ± 2.70
Stimulus threshold	42.41 ± 4.30	40.19 ± 4.58	44.33 ± 2.96	42.65 ± 4.11	44.87 ± 5.29	43.19 ± 5.03	48.31 ± 3.04	46.49 ± 3.13	44.13 ± 4.48	43.31 ± 4.75	46.43 ± 3.11	45.98 ± 3.09	43.80 ± 4.42	42.46 ± 4.81	45.33 ± 3.57	43.31 ± 3.87
Stimulus 50% max	55.38 ± 4.80	53.63 ± 5.35	60.12 ± 4.06	57.04 ± 4.11	56.20 ± 6.00	55.61 ± 6.31	59.44 ± 3.89	59.45 ± 3.82	54.20 ± 4.99	55.01 ± 5.10	58.60 ± 4.41	59.22 ± 4.29	56.04 ± 5.40	56.34 ± 5.22	58.95 ± 4.27	60.94 ± 4.75
Stimulus max	68.35 ± 5.65	67.07 ± 6.36	75.90 ± 5.84	71.42 ± 5.25	67.53 ± 7.04	68.02 ± 7.75	70.56 ± 5.00	72.41 ± 4.62	64.26 ± 5.76	66.70 ± 5.77	70.76 ± 5.80	72.46 ± 5.81	68.28 ± 6.76	70.23 ± 6.01	72.57 ± 5.28	78.57 ± 6.68

Results of predicted parameters from the sigmoid input-output relation of TEPs recorded at all points of the recruitment curve. TEPs were normalized to the homonymous maximal TEP recorded before PAS and were plotted against real stimulation intensities. This was performed separately for each subject, and averages were estimated and grouped based on time. *m*: slope parameter of the function. Mean ± SE. TA: tibialis anterior; MG: medial gastrocnemius; SOL: soleus; PL: peroneus longus; R: right; L: left. Italics indicate statistically significant differences before and after PAS.

## Abbreviations

LTD: Long-term depression
MEPs: Motor-evoked potentials
MG: Medial gastrocnemius
PAS: Paired associative stimulation
PL: Peroneus longus
SOL: Soleus
TA: Tibialis anterior
TEPs: Transspinal evoked potentials
TMS: Transcranial magnetic stimulation.
